# Comparative evaluation of explainable vision models for bone tumour detection on radiographs

**DOI:** 10.3389/fdgth.2026.1926330

**Published:** 2026-09-23

**Authors:** Daniel Jones Ortega, Khuhed Memon, Syed Saad Azhar Ali, Yasir Hafeez

**Affiliations:** 1 School of Computer Science, University of Nottingham, Nottingham, United Kingdom; 2 Department of Electronic Engineering, Mehran University of Engineering and Technology, Jamshoro, Pakistan; 3 Department of Aerospace Engineering and Interdisciplinary Research Center for Smart Mobility and Logistics, King Fahd University of Petroleum & Minerals (KFUPM), Dhahran, Saudi Arabia; 4 School of Computer and Mathematical Sciences, Faculty of Science and Engineering, University of Nottingham, Semenyih, Malaysia

**Keywords:** bone tumour detection, BTXRD, classification, deep learning, explainable AI, radiographs, segmentation, YOLOv8

## Abstract

**Introduction:**

Accurate detection of bone tumours on radiographs can be challenging because lesion appearance varies and interpretation requires specialist expertise. This study evaluates deep learning (DL) models for bone tumour localisation, segmentation, classification, and explainability using the publicly available Bone Tumour X-Ray Radiograph Dataset (BTXRD), comprising 3,746 musculoskeletal radiographs.

**Methods:**

A YOLOv8s-seg model was evaluated alongside MobileNetV3-Large, EfficientNet-B3, and ConvNeXt-Tiny classifiers trained under a common-resolution protocol. Grad-CAM, Grad-CAM++, and Layer-CAM activation maps were compared with expert tumour masks using mean Intersection over Union and a thresholded lesion-overlap measure. For the retrospective agreement analysis, EfficientNet-B3 was paired with YOLOv8s-seg. An exploratory external series of 11 tumour-positive radiographs was also examined.

**Results:**

ConvNeXt-Tiny achieved the highest binary classification accuracy (0.879), while YOLOv8s-seg achieved an overall box mAP50 of 0.676 and mask mAP50 of 0.668. The components produced concordant binary tumour-presence predictions for 605 of 750 test radiographs, corresponding to 80.7% coverage (95% confidence interval [CI]: 77.7%–83.3%). Binary accuracy within the agreed subset was 93.6% (566/605; 95% CI: 91.3%–95.2%), while the remaining 145 cases were discordant and received no consensus output.

**Discussion:**

The XAI analyses quantify spatial correspondence rather than explanation faithfulness or clinical reliability. Because the exploratory external series contained seven benign and four malignant tumour-positive radiographs and no normal radiographs, it cannot assess specificity or false-positive behaviour and is not presented as representative external validation.

## 1 Introduction

X-ray radiographs are widely used in modern medical imaging because they enable non-invasive analysis of a patient’s internal anatomy, supporting screening, diagnosis, and treatment planning (1). They serve as a first-line imaging modality for musculoskeletal evaluation due to their speed and wide availability. For conditions such as primary bone tumours, where timely diagnosis can substantially influence treatment options, their role is critical (2). This is particularly important as primary malignant bone tumours represent the third leading cause of cancer-related mortality in patients under the age of 20 (3).

Despite their clinical utility, radiographs have inherent limitations. As 2D projections, they compress complex 3D anatomy into a single plane, making subtle lesions difficult to detect. The accurate interpretation of these images requires substantial expertise. However, specialist availability is often limited, which can lead to large workloads, potentially delaying diagnosis and increasing the risk of perceptual or interpretive errors (4).

To mitigate these issues, artificial intelligence (AI), particularly deep learning (DL), has been explored as an assistive technology. Convolutional neural networks (CNNs) have shown strong performance in detecting visual patterns directly from pixel data (5). However, a significant limitation to the widespread adoption of AI in healthcare is the ”black box” nature of DL models, whose internal decision-making processes lack transparency. Explainable AI (XAI) aims to address this limitation. Techniques such as Gradient-weighted Class Activation Mapping (Grad-CAM) can produce attention heatmaps that highlight the image regions contributing to a model’s prediction (6). In medical imaging, such heatmaps can provide a visual indication of the image regions associated with a model prediction, although their clinical value depends on how reliably they reflect model behaviour.

The motivation for this research is the need for reproducible methods that support accurate bone tumour detection while allowing model outputs to be examined. Although prior work has explored DL for bone tumour detection, many studies have relied on private datasets and have provided limited quantitative evaluation of explainability methods. The public Bone Tumour X-Ray Radiograph (BTXRD) dataset (3) provides an opportunity to compare modern architectures under a common experimental setting.

The primary aim of this research is to develop and evaluate a DL pipeline for bone tumour detection on radiographs while examining the spatial behaviour of gradient-based XAI methods. The study therefore: (1) evaluates YOLOv8s-seg for lesion localisation, segmentation, and subtype classification together with three CNN backbones for binary tumour-presence classification; (2) assesses the contribution of contextual metadata to classification performance; (3) examines model performance across anatomical sites and lesion-size groups; (4) evaluates Grad-CAM, Grad-CAM++, and Layer-CAM against tumour annotations; and (5) examines agreement between the CNN and YOLOv8s-seg components, followed by an exploratory assessment on a small external tumour-positive case series.

## 2 Related work

### 2.1 Deep learning in medical imaging

Deep learning methods, particularly convolutional neural networks (CNNs), are widely used in medical image analysis. CNNs learn hierarchical visual features directly from image data, reducing the need for manual feature extraction and supporting tasks such as image classification and localisation (5).

### 2.2 Deep learning for bone tumour detection

Several studies have applied DL to bone tumour detection on plain radiographs. Li et al. (7) trained a YOLOv5-based pipeline to classify bone tumours, achieving a mean Intersection over Union (IoU) of 0.68 and outperforming one radiologist on intermediate lesions. Sen et al. (8) developed a YOLOv7 detector on a dataset of 1,978 X-rays, achieving 71.65% accuracy and demonstrating the feasibility of bounding-box localisation. Rajath et al. (9) proposed a pipeline that fine-tuned multiple CNNs on over 6,000 CT scans, with a DenseNet model achieving 94% accuracy. These studies demonstrate the feasibility of DL for bone tumour imaging, but differences in datasets and limited quantitative explainability assessment make direct comparison difficult.

Recent work has increasingly incorporated XAI into bone-imaging pipelines. Studies have explored interpretable deep learning for bone cancer diagnosis (10), combined pre-trained classifiers with methods such as Grad-CAM and SHAP (11), and developed explainable CNN-based (12) and multimodal (13) frameworks. However, these studies primarily focus on binary classification, broader bone-radiology tasks, or complex multimodal imaging. This motivates further evaluation of localisation, classification, and explainability within a common radiograph dataset.

### 2.3 Explainable AI in medical imaging

While DL models offer high performance, their intricate decision-making process makes it difficult to interpret why a specific prediction was made. Explainable AI (XAI) seeks to develop methods that make AI-driven decisions more transparent and interpretable. In computer vision, a primary XAI approach is to produce visual representations of a model’s attention, often referred to as saliency maps or heatmaps.

Class Activation Mapping (CAM) enabled the identification of important image regions using feature maps but was limited to specific CNN architectures (14). To address this limitation, Gradient-weighted Class Activation Mapping (Grad-CAM) was developed (6). Unlike CAM, Grad-CAM is more flexible, using the gradients of the target class to weight feature maps, allowing it to be applied across a wider range of CNN architectures. Nonetheless, Grad-CAM can struggle to highlight small features accurately. Newer advances such as Grad-CAM++ (15), which uses refined gradient weighting, and Layer-CAM (16), which integrates information from multiple network layers, have been developed to create more precise and higher-resolution heatmaps. In medical imaging, these heatmaps can be compared with annotated pathology to examine whether model activation is spatially associated with the region of interest. However, a recent systematic review found that visual explanation methods remain dominant in diagnostic radiology and highlighted the need for stronger evaluation of explanation quality, clinical usefulness, and the needs of healthcare professionals (17).

### 2.4 Research gaps

Several limitations remain in the current literature. Bone tumour studies frequently use private datasets, limiting reproducibility and direct comparison. Quantitative evaluation of XAI is also less common than visual inspection, and studies often focus on either classification or localisation rather than evaluating complementary outputs within the same dataset. The public BTXRD dataset (3) supports reproducible comparison across these tasks. Recent reviews similarly identify the need for larger validation datasets, interpretable methods, and clinically relevant evaluation of decision-support systems (18).

## 3 Materials and methods

This study uses the publicly available BTXRD dataset (3) to evaluate a dual-component pipeline combining YOLOv8s-seg (19) for lesion localisation and segmentation with a CNN classification framework. The CNN component produces an image-only output and a fused output incorporating contextual metadata, and also provides the image representations used for the XAI analysis.

### 3.1 BTXRD dataset

The Bone Tumour X-ray Radiograph (BTXRD) dataset (3) is a publicly available, multi-centre collection of 3,746 bone radiographs. The dataset contains 1,879 normal images and 1,867 images with confirmed primary bone tumours, comprising 1,525 benign and 342 malignant cases. Tumour cases include expert-annotated segmentation masks and corresponding bounding boxes, together with image-level labels and available contextual metadata.

### 3.2 Data preparation and preprocessing

Images were resized with padding to preserve aspect ratio, using the input resolution specified for each model. Contrast-Limited Adaptive Histogram Equalisation (CLAHE) was applied to enhance local contrast, followed by image normalisation. Geometric and photometric augmentations were applied to the training set using the Albumentations library (20). For the CNN experiments, the training loader used seeded shuffling without replacement, such that each training image was sampled once per epoch. Class-balanced oversampling was not used. The contextual inputs comprised age, the dataset-recorded gender variable, and radiographic projection indicators. Gender was encoded as a binary variable (female=1,male=0), while frontal, lateral, and oblique projection were represented as binary indicators. Age was standardised using the mean and standard deviation calculated from the training partition. These contextual variables were available for all included radiographs, so no missing-value imputation was required. All preprocessing statistics derived from the training partition were applied unchanged to the validation and test partitions.

### 3.3 Dual-component model design

A dual-component design was used to evaluate complementary localisation and classification outputs (Figure 1). The first component uses YOLOv8s-seg to localise and segment tumour regions while assigning benign or malignant subtype labels. The second component performs independent binary tumour-presence classification and provides the image features used to generate Grad-CAM-based activation maps.

**Figure 1:**
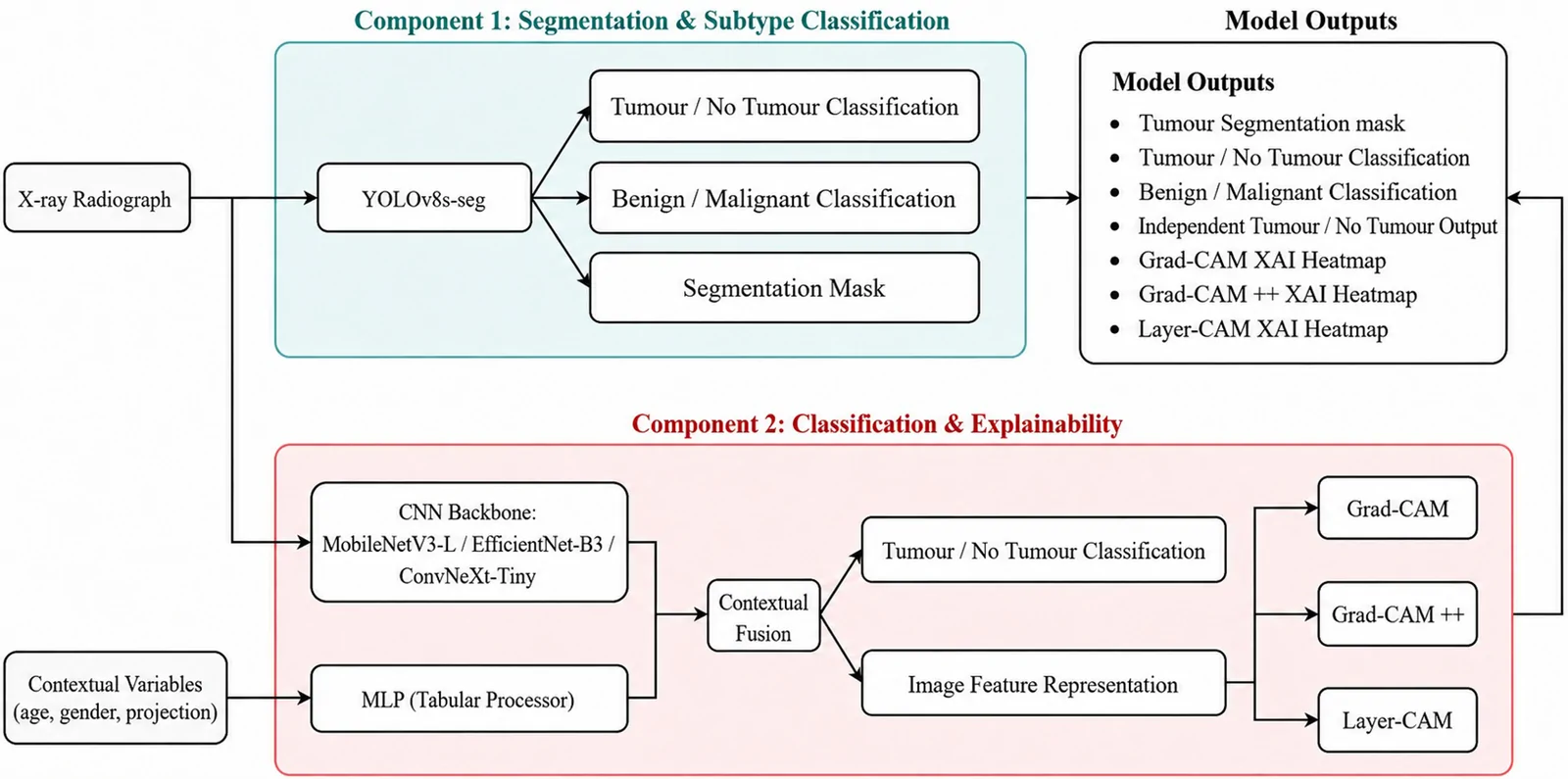
Overview of the dual-component design combining YOLOv8s-seg localisation and segmentation with CNN-based tumour-presence classification, contextual fusion, and XAI analysis.

#### 3.3.1 Component 1: segmentation and subtype classification

YOLOv8s-seg (19) was used because it supports lesion detection, instance segmentation, and class assignment within a single architecture. This matched the BTXRD annotations, which provide both lesion masks and bounding boxes. The model was trained to segment tumour regions and assign benign or malignant subtype labels. Images without tumour annotations were used as no-tumour examples. The model was initialised from COCO-pretrained YOLOv8 weights (21) and jointly optimised for classification, bounding-box localisation, and segmentation.

#### 3.3.2 Component 2: classification and explainability

Three CNN backbones were compared for binary tumour-presence classification and XAI: MobileNetV3-Large (22), EfficientNet-B3 (23), and ConvNeXt-Tiny (24). Each architecture produced an image-only classification output and a fused classification output incorporating contextual metadata.

The final feature map from the image encoder was reduced through global average pooling. The resulting image feature vector was passed directly to a linear image-only classification head. The five-dimensional contextual vector comprised standardised age, encoded gender, and frontal, lateral, and oblique radiographic-projection indicators. This vector was processed by a two-layer multilayer perceptron with dimensions 32 and 16, ReLU activations, layer normalisation, and dropout of 0.10. The resulting contextual embedding was concatenated with the pooled image features and passed to a separate fused classification head. The image-only and fused classification heads were trained jointly over the same shared image encoder.

### 3.4 Training and evaluation protocol

The BTXRD dataset was partitioned at the image level using fixed image-identifier manifests comprising 2,396 training images, 600 validation images, and 750 test images, corresponding approximately to a 64/16/20 split. Each manifest contained unique image identifiers, and pairwise checks confirmed that no image appeared in more than one partition. The same partitions were used for YOLOv8s-seg and all three CNN classification architectures. The validation partition was used during model development and model selection, while the test partition was reserved for final evaluation.

Patient and examination identifiers suitable for grouping related radiographs were not available in the dataset used in this study. Patient-level separation could therefore not be verified, although image-level overlap between the partitions was excluded. Accordingly, all reported performance estimates are image-level rather than patient-level results.

The implementation was carried out in PyTorch, with Ultralytics used for the YOLOv8s-seg model and timm used for the CNN backbone implementations. The AdamW optimiser was used across the classification experiments. Model training was performed in Google Colab using an NVIDIA L4 GPU with 24 GB of GPU memory.

For the segmentation component, YOLOv8s-seg was trained for 100 epochs using an image size of 640 pixels, a batch size of 16, a learning rate of $1\times 10^{-3}$, and a weight decay of $5\times 10^{-4}$.

To provide a controlled comparison of the classification architectures, MobileNetV3-Large, EfficientNet-B3, and ConvNeXt-Tiny were trained using a common input resolution of 512×512 pixels. All three architectures used the same fixed data partitions, preprocessing and augmentation framework, random seed, optimisation schedule, and validation-based checkpoint-selection procedure.

Training was conducted in two stages using ImageNet-pretrained backbones. During the first stage, the image encoder was frozen for 15 epochs while the remaining layers were optimised using AdamW with a maximum learning rate of $5\times 10^{-4}$. During the second stage, the complete network was fine-tuned for 25 epochs. The maximum learning rate was $2.5\times 10^{-5}$ for the pretrained encoder and $1\times 10^{-4}$ for the remaining layers. A OneCycle learning-rate schedule and a weight decay of $2\times 10^{-4}$ were used. For each architecture, the checkpoint achieving the highest fused ROC-AUC on the validation partition was retained for evaluation.

Batch sizes were adjusted to accommodate architecture-specific GPU memory requirements. MobileNetV3-Large used batch sizes of 8 during frozen-encoder training and 4 during full-network fine-tuning. EfficientNet-B3 and ConvNeXt-Tiny used batch sizes of 4 and 2, respectively. Batch sizes therefore differed by architecture and are reported explicitly for reproducibility.

The contribution of contextual metadata was assessed by comparing the image-only and fused outputs from each architecture on the same held-out test images. Both outputs were produced by jointly trained classification heads sharing the same image encoder.

All classifier decision thresholds used for test-set evaluation were selected on the validation partition and then fixed before test evaluation. Threshold-independent discrimination was assessed using ROC-AUC, while threshold-dependent performance included accuracy, sensitivity, precision, and F1-score. Image-only and fused outputs were compared using 2,000 paired image-level bootstrap resamples and exact McNemar tests for paired correctness; bootstrap probability values were adjusted across the three architectures using the Holm procedure.

This comparison therefore estimates the incremental contribution of contextual metadata within each architecture rather than comparing independently trained models.

### 3.5 Anatomical-site and lesion-size subgroup analysis

Anatomical-site subgroup analyses were conducted on the tumour-positive test radiographs using the site indicators provided in the BTXRD dataset. These indicators were not mutually exclusive, and cases without a recorded site indicator were excluded from the corresponding subgroup analysis. For YOLOv8s-seg and the CNN classifiers, tumour-presence sensitivity was calculated within each anatomical-site subgroup. The CNN analyses used the primary fused outputs and their validation-derived decision thresholds. Wilson 95% confidence intervals were calculated for the site-specific sensitivity estimates.

An exploratory lesion-size subgroup analysis was conducted on the tumour-positive radiographs. For each tumour-positive radiograph with an expert polygon annotation, relative lesion area was defined as the number of pixels within the union of the annotated tumour polygons divided by the total number of pixels in the original radiograph. To avoid defining the subgroups using the test data, the 33rd and 67th percentiles of relative lesion area were calculated from the 1,174 annotated tumour-positive radiographs in the training partition. These produced fixed cut-offs of 0.003140 and 0.020829. Lesions were therefore categorised as small (≤0.003140), medium (>0.003140 and ≤0.020829), or large (>0.020829). These cut-offs were applied unchanged to all 384 tumour-positive radiographs in the held-out test partition.

For YOLOv8s-seg, tumour-presence sensitivity and mean mask IoU were calculated within each lesion-size group. For the CNN classifiers, the primary fused outputs and their validation-derived decision thresholds were used to calculate tumour-presence sensitivity with Wilson 95% confidence intervals. Because the subgroups contained only tumour-positive radiographs, this analysis focused on missed tumour detection rather than specificity or benign-vs.-malignant subtype errors.

### 3.6 Dual-component agreement analysis

Agreement between EfficientNet-B3 and YOLOv8s-seg was evaluated retrospectively on the held-out test partition. EfficientNet-B3 was used for this retrospective analysis because it retained strong classification performance while showing substantially higher thresholded lesion-overlap rates than ConvNeXt-Tiny. The fused EfficientNet-B3 output produced a continuous tumour-presence score. A decision threshold of 0.787598 was selected using the validation partition and was subsequently applied unchanged to the held-out test partition.

YOLOv8s-seg inference was performed at an image size of 640 pixels using a detection-retrieval confidence threshold of 0.001, a non-maximum-suppression IoU threshold of 0.70, and a maximum of 300 detections per radiograph. An image-level tumour-presence threshold of 0.094897 was selected exclusively on the 600-image validation partition by maximising balanced accuracy and was subsequently fixed and applied unchanged to the held-out test partition.

For each radiograph, the highest benign or malignant detection score was retained. A score at or above the selected threshold produced a tumour-positive output; otherwise, the component produced a no-tumour output. When multiple eligible detections were present, the class associated with the highest-scoring detection was used for the benign-vs.-malignant subtype output.

A case was considered agreed when the thresholded EfficientNet-B3 and YOLOv8s-seg outputs both indicated tumour presence or both indicated no tumour. All other cases were considered discordant. Agreement was assessed only at the binary tumour-presence level and did not require agreement on benign-vs.-malignant subtype.

Coverage was defined as the proportion of test radiographs receiving concordant binary predictions. Selective accuracy was defined as binary tumour-presence accuracy among these agreed cases. Discordant cases did not receive a consensus output. Wilson 95% confidence intervals were calculated for coverage and selective accuracy. Results were additionally stratified by the reference no-tumour, benign, and malignant categories; within the benign and malignant strata, selective accuracy refers only to binary tumour-presence classification and not to subtype classification.

The values used by these decision rules are raw neural-network or detector scores. They were not interpreted as calibrated clinical probabilities. This analysis represents a single retrospective agreement rule rather than a prospectively validated abstention or clinical triage system.

### 3.7 Calibration assessment

A descriptive calibration assessment was conducted for the primary fused outputs of MobileNetV3-Large, EfficientNet-B3, and ConvNeXt-Tiny across the 750 test radiographs. Calibration was assessed using the Brier score, expected calibration error (ECE) calculated from 10 equal-width score intervals, and calibration slope; an ideally calibrated output has a slope of 1. This analysis did not alter the original model scores or the validation-derived decision thresholds. YOLOv8s-seg detector confidence was treated as an operating score rather than as an estimated clinical probability.

### 3.8 Explainable AI methodology and evaluation

An XAI analysis was conducted using Grad-CAM (6), Grad-CAM++ (15), and Layer-CAM (16). The analysis was restricted to the image-only classification output because the fused output also incorporated non-imaging contextual variables that cannot be represented spatially in an image heatmap. For all three architectures, the target was therefore the tumour-positive image-only classification logit.

For each architecture, the activation maps were generated from the final spatial feature representation of the image encoder, immediately before global average pooling and classification. This corresponded to the final convolutional feature representation for MobileNetV3-Large and EfficientNet-B3 and the final spatial feature representation for ConvNeXt-Tiny. The same target representation was used for Grad-CAM, Grad-CAM++, and Layer-CAM within each architecture.

The quantitative analysis included all 384 tumour-positive radiographs in the held-out test partition for which expert polygon annotations were available. No annotated tumour-positive test images were excluded. Cases were included irrespective of whether the image-only classifier produced a true-positive or false-negative thresholded prediction, because the analysis evaluated the spatial distribution of evidence associated with the tumour-positive logit rather than only correctly classified cases.

Images and reference masks were resized to the common 512×512 model input space using the same aspect-ratio-preserving padding procedure. Image interpolation was bilinear, whereas reference masks were resized using nearest-neighbour interpolation. Each generated activation map was resized to the model input resolution and independently min–max normalised to the interval [0,1].

Two spatial-alignment measures were calculated. For the Intersection over Union analysis, the pixels with the highest 15% of normalised CAM activation were selected to form a binary activation region. Mean IoU was then calculated between this region and the corresponding expert tumour mask.

A thresholded lesion-overlap rate was also calculated as the proportion of tumour-positive cases in which at least one pixel within the expert tumour mask had a normalised CAM activation of at least 0.5. This is not a conventional pointing-game metric because it does not require the single maximum-activation pixel to fall inside the lesion.

Mean-IoU 95% confidence intervals were estimated using 2,000 image-level bootstrap resamples. Wilson 95% confidence intervals were calculated for the lesion-overlap rates. These metrics quantify spatial correspondence between activation maps and expert tumour masks. They do not measure causal explanation faithfulness, diagnostic reliability, or clinical usefulness.

For qualitative error analysis, one false-positive and one false-negative EfficientNet-B3 image-only prediction were selected from the held-out test set using a score-based selection rule. The false-positive example was the highest-scoring false positive, whereas the false-negative example was the lowest-scoring false negative according to the image-only tumour score. Grad-CAM, Grad-CAM++, and Layer-CAM were generated for both cases using the same XAI procedure described above. These examples were included for qualitative illustration and were not intended to represent the full distribution of classification errors.

### 3.9 External case-series reference data

The external case series was provided by clinical collaborators and comprised 11 radiographs paired with binary segmentation masks, including seven benign and four malignant cases. The radiographs were supplied in JPEG format and the corresponding masks in PNG format. Dr. Shahabuddin Siddiqui of the Advanced Diagnostic Center and Advanced International Hospital, Islamabad, Pakistan, provided and annotated the cases. The diagnostic labels and segmentation masks were used as supplied for the external case-level evaluation. No additional re-annotation or adjudication was performed by the present study team.

## 4 Results

### 4.1 Dataset characteristics

The BTXRD dataset was closely balanced for tumour presence, with 1,867 tumour-positive and 1,879 no-tumour radiographs. Benign cases were more common than malignant cases, and anatomical-site representation was uneven, providing context for the subgroup analyses reported below.

### 4.2 YOLOv8s-seg performance (component 1)

#### 4.2.1 Overall localisation, segmentation, and classification performance

YOLOv8s-seg was evaluated for lesion localisation, segmentation, and three-class image-level classification (Table 1). Overall box mAP50 was 0.676 and mask mAP50 was 0.668. On the three-class classification task, accuracy was 0.852 and macro F1-score was 0.834. For binary tumour presence, accuracy was 0.869, sensitivity was 0.799, and specificity was 0.943. Evaluation used the held-out image-level test partition of 750 radiographs, comprising 366 no-tumour and 384 tumour-positive images.

**Table 1:** Overall YOLOv8s-seg localisation, segmentation, and classification performance.

Category	Localisation and segmentation				Image-level classification		
	Box mAP50	Box mAP50-95	Mask mAP50	Mask mAP50-95	Precision	Recall	F1-score
Overall	0.676	0.380	0.668	0.356	0.871	0.810	0.834
Benign	0.639	0.356	0.630	0.327	0.895	0.781	0.834
Malignant	0.714	0.404	0.705	0.385	0.902	0.705	0.791

Overall classification precision, recall, and F1-score are macro-averaged across the three image-level classes (No Tumour, Benign, and Malignant). Overall three-class classification accuracy was 0.852.

The confusion matrix in Figure 2 shows that most errors involved missed tumour cases: 61 benign and 16 malignant radiographs were classified as “No Tumour”. This pattern motivated further analysis by anatomical site and lesion size.

**Figure 2:**
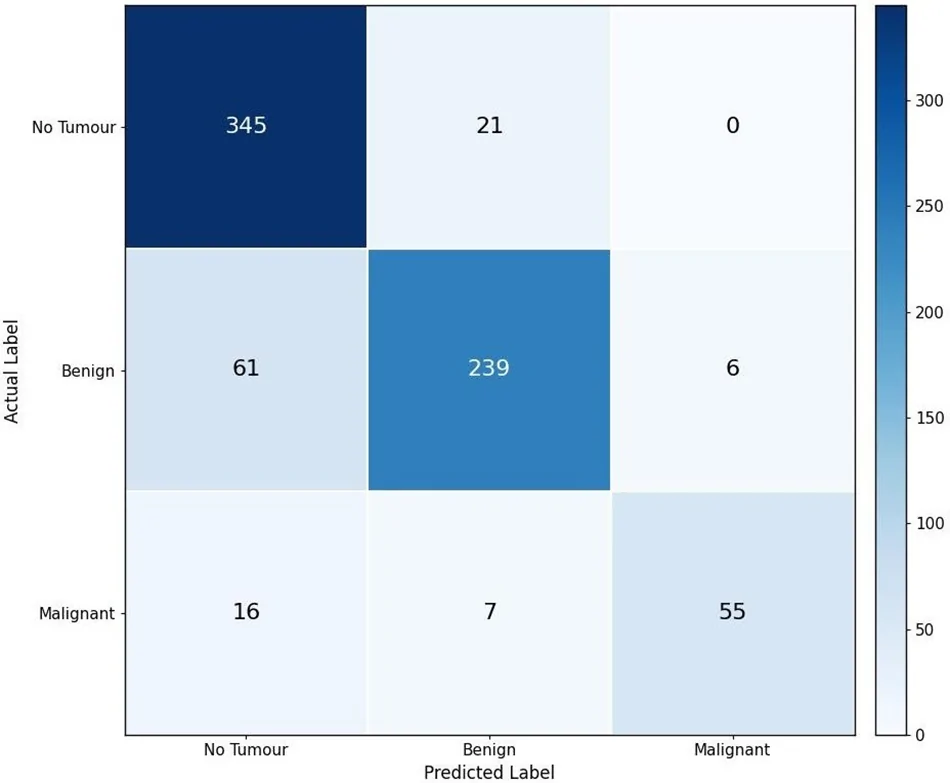
Confusion matrix for the YOLOv8s-seg model on the three-class classification task.

#### 4.2.2 Performance by anatomical site and lesion size

Tumour-detection sensitivity varied across anatomical sites (Table 2), with higher observed sensitivity for the tibia, fibula, and ulna and lower sensitivity for the hip bone and hand.

**Table 2:** YOLOv8s-seg tumour-detection sensitivity by anatomical site.

Metric	Hand	Ulna	Radius	Humerus	Foot	Tibia	Fibula	Femur	Hip bone
Cases	17	14	20	63	12	123	43	114	14
Sensitivity	0.647	0.857	0.750	0.841	0.667	0.886	0.860	0.833	0.500
95% CI, lower	0.413	0.601	0.531	0.732	0.391	0.818	0.727	0.754	0.268
95% CI, upper	0.827	0.960	0.888	0.911	0.862	0.931	0.934	0.891	0.732

Lesion-size analysis showed increasing tumour-detection sensitivity from 0.775 (95% CI: 0.692–0.841) for small lesions (n=120) and 0.782 (95% CI: 0.707–0.842) for medium lesions (n=142) to 0.844 (95% CI: 0.770–0.898) for large lesions (n=122). Mean mask IoU also increased from 0.462 for small lesions to 0.571 for medium lesions and 0.605 for large lesions. These descriptive results indicate that smaller lesions were more frequently missed and were more difficult to delineate.

### 4.3 Classification model performance (component 2)

Whereas YOLOv8s-seg provides lesion localisation, segmentation, and subtype information, the second component provides an independent whole-image assessment of tumour presence and the image features used for XAI. MobileNetV3-Large, EfficientNet-B3, and ConvNeXt-Tiny were compared for this task.

#### 4.3.1 Overall diagnostic performance

The controlled comparison of the three classification architectures is summarised in Table 3. All models were evaluated on the same 750-image test partition after training under the common 512×512 protocol. The primary analysis used the fused output, with output-specific decision thresholds selected exclusively on the validation partition and applied unchanged to the test set. Precision, sensitivity, and F1-score refer to the tumour-positive class. Image-only ROC-AUC and accuracy are also reported to support the within-model assessment of contextual metadata. ConvNeXt-Tiny achieved the strongest overall classification performance, while MobileNetV3-Large and EfficientNet-B3 showed lower but broadly comparable performance.

**Table 3:** CNN classification performance for fused and image-only outputs across 750 test radiographs.

Model	Fused output					Image-only output	
	Precision	Sensitivity	F1	ROC-AUC	Accuracy	ROC-AUC	Accuracy
MobileNetV3-Large	0.887	0.773	0.826	0.903	0.833	0.899	0.833
EfficientNet-B3	0.875	0.786	0.829	0.918	0.833	0.915	0.833
ConvNeXt-Tiny	0.925	0.831	0.875	0.942	0.879	0.942	0.877

Figure 3 further illustrates the differences in classification errors across the three architectures. ConvNeXt-Tiny produced the fewest false-positive and false-negative predictions, with 26 no-tumour radiographs classified as tumour and 65 tumour-positive radiographs classified as no tumour. MobileNetV3-Large and EfficientNet-B3 showed more missed tumour cases, with 87 and 82 false negatives, respectively. This pattern is consistent with the higher sensitivity and overall accuracy observed for ConvNeXt-Tiny.

**Figure 3:**
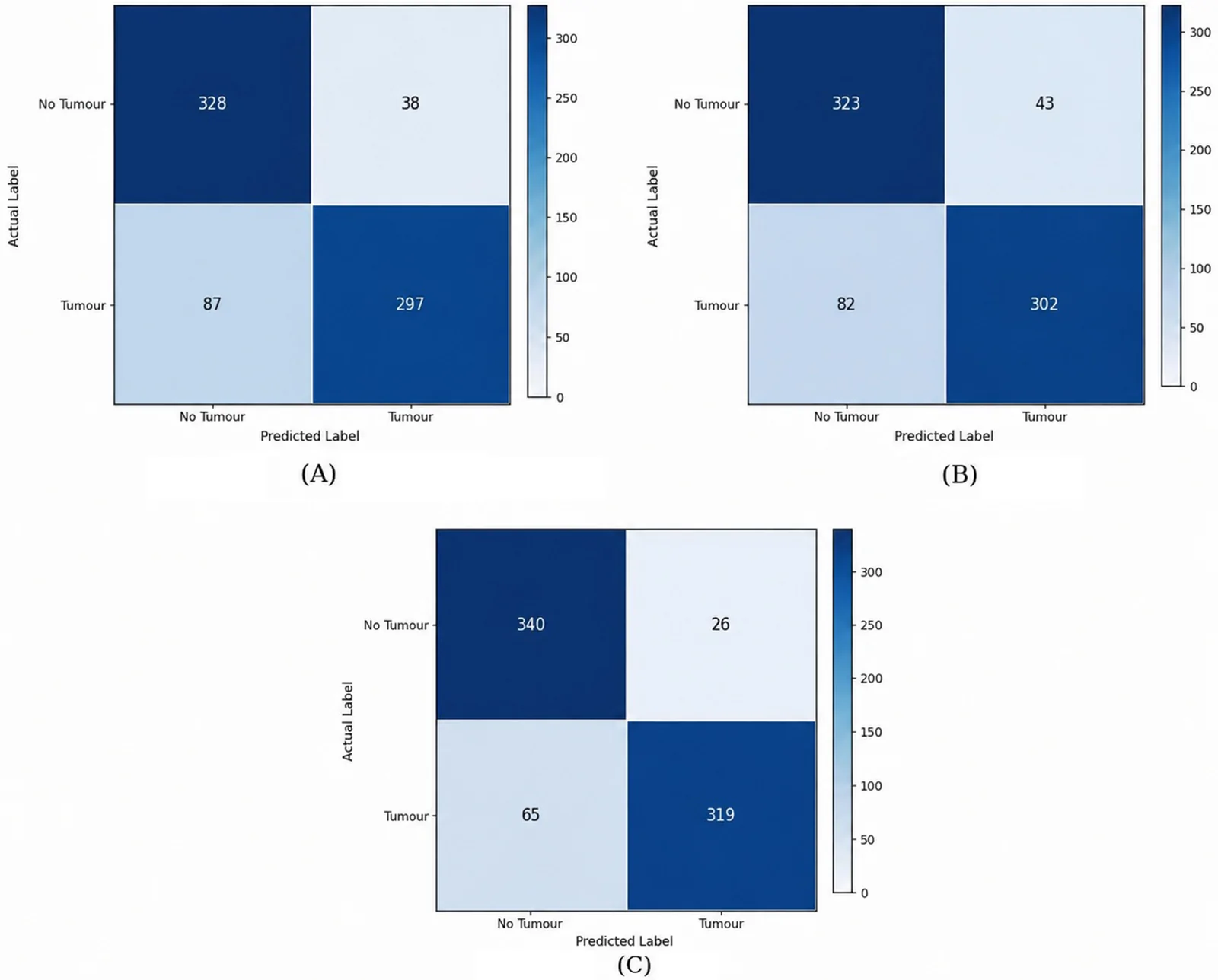
Comparative confusion matrices for **(A)** MobileNetV3-Large, **(B)** EfficientNet-B3, and **(C)** ConvNeXt-Tiny.

#### 4.3.2 Contribution of contextual metadata

The image-only and fused outputs were compared to determine whether age, dataset-recorded gender, and radiographic projection added predictive information beyond the radiograph (Table 3). Metadata fusion produced only small changes in ROC-AUC and did not improve accuracy for MobileNetV3-Large or EfficientNet-B3; ConvNeXt-Tiny showed a similarly small change in both measures. Paired bootstrap analysis showed small fused-minus-image-only ROC-AUC differences for MobileNetV3-Large (+0.0039, 95% CI: 0.0017–0.0065), EfficientNet-B3 (+0.0025, 95% CI: 0.0006–0.0047), and ConvNeXt-Tiny (+0.0004, 95% CI: −0.0007–0.0014). Overall, contextual metadata provided limited incremental predictive value.

#### 4.3.3 Calibration of model scores

In addition to classification performance, model calibration was assessed to examine how closely the model scores corresponded to observed tumour outcomes (Table 4). ConvNeXt-Tiny had the lowest Brier score and ECE, while calibration slopes were substantially below the ideal value of 1 for all three architectures. The raw classifier scores should therefore not be interpreted as calibrated probabilities of tumour presence.

**Table 4:** Descriptive calibration assessment of the primary fused CNN outputs across 750 test radiographs.

Model	Brier score	10-bin ECE	Calibration slope (95% CI)
MobileNetV3-Large	0.1517	0.1456	0.215 (0.188–0.243)
EfficientNet-B3	0.1492	0.1467	0.183 (0.161–0.205)
ConvNeXt-Tiny	0.1083	0.1052	0.192 (0.170–0.214)

#### 4.3.4 CNN classification performance by anatomical site and lesion size

Classification performance was also examined across anatomical sites and lesion-size groups. Exploratory site-specific tumour-presence sensitivity varied across anatomical regions (Table 5).

**Table 5:** Exploratory tumour-presence sensitivity by anatomical site for the primary fused CNN outputs.

Model	Metric	Hand	Ulna	Radius	Humerus	Foot	Tibia	Fibula	Femur	Hip bone
	Cases	17	14	20	63	12	123	43	114	14
MobileNetV3-Large	Sensitivity	0.412	0.571	0.700	0.746	0.667	0.821	0.814	0.851	0.857
	95% CI, lower	0.216	0.326	0.481	0.627	0.391	0.744	0.674	0.774	0.601
	95% CI, upper	0.640	0.786	0.855	0.837	0.862	0.879	0.903	0.905	0.960
EfficientNet-B3	Sensitivity	0.412	0.643	0.650	0.746	0.583	0.846	0.814	0.886	0.929
	95% CI, lower	0.216	0.388	0.433	0.627	0.320	0.771	0.674	0.815	0.685
	95% CI, upper	0.640	0.837	0.819	0.837	0.807	0.899	0.903	0.932	0.987
ConvNeXt-Tiny	Sensitivity	0.706	0.643	0.800	0.873	0.583	0.902	0.860	0.860	0.857
	95% CI, lower	0.469	0.388	0.584	0.769	0.320	0.837	0.727	0.784	0.601
	95% CI, upper	0.867	0.837	0.919	0.934	0.807	0.943	0.934	0.912	0.960

The effect of lesion size on binary tumour-presence classification was also examined using the primary fused output of each CNN architecture (Table 6).

**Table 6:** Exploratory tumour-presence sensitivity by lesion-size group for the primary fused CNN outputs.

Model	Lesion size	Cases	Sensitivity (95% CI)
MobileNetV3-Large	Small	120	0.617 (0.527–0.699)
	Medium	142	0.782 (0.707–0.842)
	Large	122	0.918 (0.856–0.955)
EfficientNet-B3	Small	120	0.625 (0.536–0.706)
	Medium	142	0.803 (0.730–0.860)
	Large	122	0.926 (0.866–0.961)
ConvNeXt-Tiny	Small	120	0.675 (0.587–0.752)
	Medium	142	0.852 (0.784–0.901)
	Large	122	0.959 (0.908–0.982)

Sensitivity was lowest for small lesions and highest for large lesions across all three classifiers, indicating that smaller tumour-positive cases were more frequently missed. Lesion-size cut-offs were derived from the tumour-positive training partition and applied unchanged to the test partition.

#### 4.3.5 Explainable AI (XAI) evaluation

To complement the classification results, the XAI analysis examined whether the regions highlighted by each model overlapped with the annotated tumour. Grad-CAM, Grad-CAM++, and Layer-CAM were evaluated across all 384 tumour-positive test radiographs, including image-only false-negative predictions. Mean IoU was low across all model–method combinations (Table 7). MobileNetV3-Large and EfficientNet-B3 showed broadly similar spatial alignment, whereas ConvNeXt-Tiny produced substantially lower lesion-overlap rates despite achieving the strongest overall classification performance.

**Table 7:** Spatial alignment of XAI activation maps with expert tumour masks across 384 tumour-positive test radiographs.

Model	XAI method	Mean IoU (95% CI)	Thresholded lesion-overlap rate, % (95% CI)
MobileNetV3-Large	Grad-CAM	0.0906 (0.0784–0.1033)	77.9 (73.4–81.7)
	Grad-CAM++	0.0942 (0.0819–0.1077)	83.6 (79.6–87.0)
	Layer-CAM	0.0948 (0.0821–0.1075)	87.0 (83.2–90.0)
EfficientNet-B3	Grad-CAM	0.0919 (0.0794–0.1052)	79.4 (75.1–83.2)
	Grad-CAM++	0.0893 (0.0774–0.1019)	80.7 (76.5–84.4)
	Layer-CAM	0.0902 (0.0781–0.1024)	81.2 (77.0–84.8)
ConvNeXt-Tiny	Grad-CAM	0.0709 (0.0616–0.0806)	23.7 (19.7–28.2)
	Grad-CAM++	0.0680 (0.0593–0.0773)	24.2 (20.2–28.7)
	Layer-CAM	0.0640 (0.0560–0.0723)	23.4 (19.5–27.9)

These measurements describe spatial correspondence between activation maps and tumour annotations. They do not establish causal faithfulness, clinical reliability, or suitability for precise lesion localisation.

For the subsequent retrospective agreement analysis, EfficientNet-B3 was used because it retained strong classification performance while showing substantially higher thresholded lesion-overlap rates than ConvNeXt-Tiny.

Illustrative EfficientNet-B3 classification errors are provided in Supplementary Figure S1. Panel A shows a false-positive prediction in a no-tumour radiograph, where Grad-CAM, Grad-CAM++, and Layer-CAM show strong activation over the distal foot despite the absence of an annotated tumour. Panel B shows a false-negative prediction in a tumour-positive radiograph, where the activation maps overlap part of the annotated tumour region even though the image-only classifier produced a negative output. These examples show that spatial activation patterns do not necessarily correspond directly to the final thresholded classification outcome.

### 4.4 Dual-component case examples and agreement analysis

After evaluating the two components separately, their outputs were compared to determine how often they agreed on tumour presence. The analysis used the EfficientNet-B3–YOLOv8s-seg configuration, with agreement defined only at the binary tumour-presence level. Scores shown in the case examples are raw model outputs rather than calibrated probabilities.

Figures 4, 5 show concordant and discordant examples, respectively. They were selected to illustrate the two agreement outcomes and were not intended to represent the overall distribution of test-set performance. In the concordant case, both components predicted tumour presence. In the discordant case, YOLOv8s-seg did not produce a tumour detection while EfficientNet-B3 predicted tumour presence; under the agreement rule this case received no consensus output.

**Figure 4:**
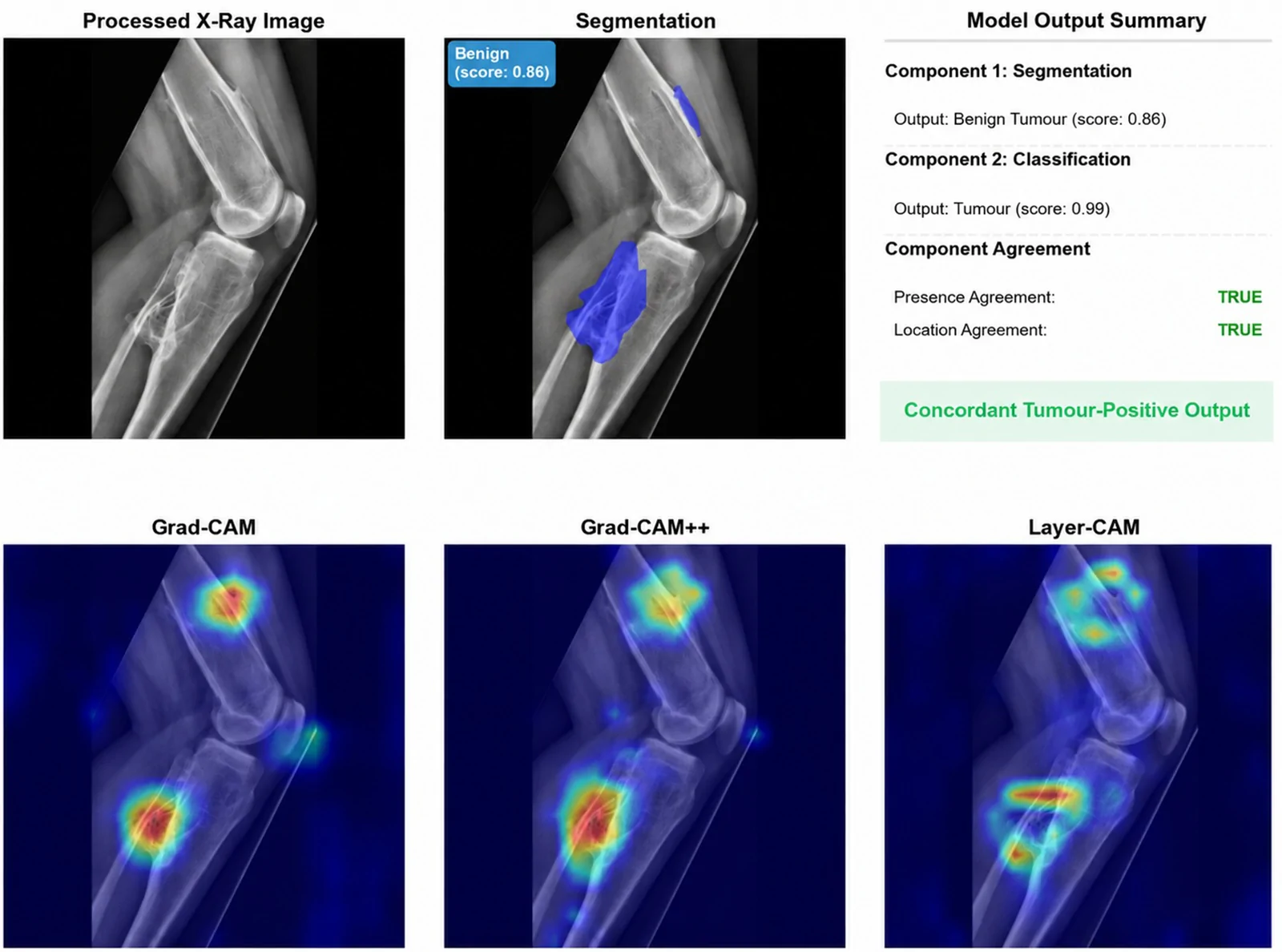
Concordant example from the agreement-based analysis. Both the EfficientNet-B3 and YOLOv8s-seg components predicted tumour presence. The underlying radiographs were obtained directly from the BTXRD dataset by Yao et al. (https://doi.org/10.6084/m9.figshare.27865398), licensed under CC BY 4.0.

**Figure 5:**
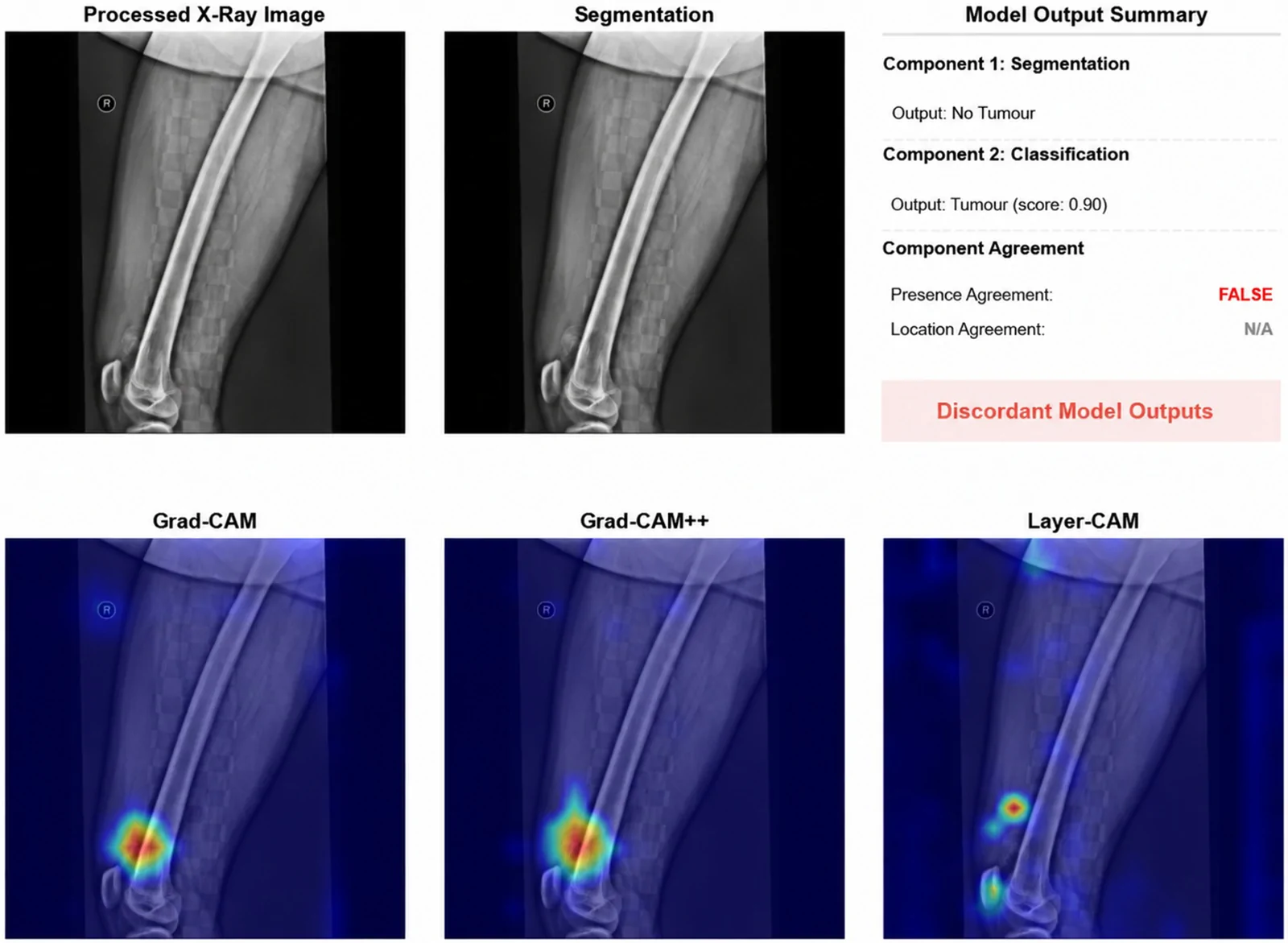
Discordant example from the agreement-based analysis. YOLOv8s-seg did not produce a tumour detection while EfficientNet-B3 predicted tumour presence; under the agreement rule, the case was discordant and received no consensus output. The underlying radiographs were obtained directly from the BTXRD dataset by Yao et al. (https://doi.org/10.6084/m9.figshare.27865398), licensed under CC BY 4.0.

Figure 4 shows a tumour-positive case in which both components independently identified tumour presence. YOLOv8s-seg localised and segmented the visible lesion, while EfficientNet-B3 produced a tumour-positive classification. The activation maps also emphasised regions around the radiographic abnormality, although the highlighted areas extended beyond the segmented lesion.

Figure 5 shows a contrasting discordant case. YOLOv8s-seg produced no tumour detection, whereas EfficientNet-B3 generated a tumour-positive output. The activation maps show concentrated classifier responses despite the absence of a corresponding segmentation, illustrating the different outputs produced by the two components when their binary predictions disagree.

The overall agreement-analysis results are summarised in Table 8. The two components agreed on 80.7% of the 750 test radiographs, with a selective accuracy of 93.6% within the agreed subset. The remaining 145 cases were discordant and received no consensus output; selective accuracy therefore should not be interpreted as overall test-set accuracy.

**Table 8:** Retrospective agreement-based analysis for the EfficientNet-B3 and YOLOv8s-seg configuration.

Reference group	Cases	Coverage, % (95% CI)	Selective accuracy, % (95% CI)
Overall	750	80.7 (77.7–83.3)	93.6 (91.3–95.2)
No tumour	366	83.6 (79.5–87.0)	99.3 (97.6–99.8)
Benign	306	76.5 (71.4–80.9)	85.9 (80.9–89.8)
Malignant	78	83.3 (73.5–90.0)	93.8 (85.2–97.6)

Stratified results showed that selective accuracy was highest for no-tumour radiographs (99.3%) and lower for benign (85.9%) and malignant (93.8%) cases, while coverage was lowest for benign cases (76.5%). These findings reinforce the importance of interpreting selective accuracy together with coverage.

### 4.5 Exploratory external tumour-positive case series

The external series described in Section 3.9 comprised seven benign and four malignant tumour-positive radiographs and no normal radiographs. It was therefore treated as a descriptive case series rather than representative external validation and could not estimate specificity or false-positive behaviour.

#### 4.5.1 Illustrative external cases

Figure 6 presents one benign case, one malignant case, and one case with discordant component outputs, selected to illustrate different observed model behaviours.

**Figure 6:**
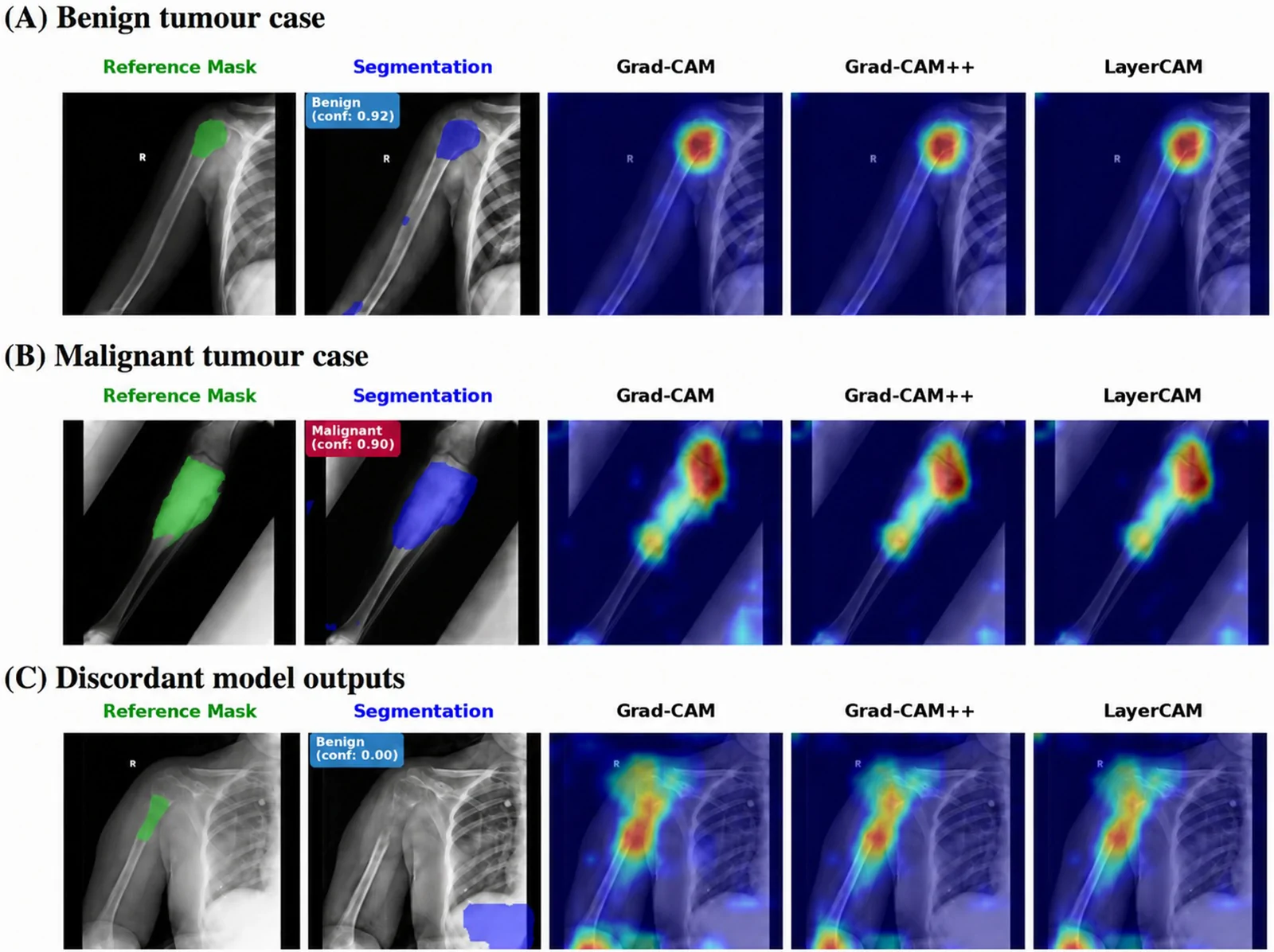
Illustrative cases from the exploratory external tumour-positive series. **(A)** Benign case with predicted segmentation and reference mask. **(B)** Malignant case with predicted segmentation and XAI heatmap. **(C)** Discordant case with segmentation not aligned to the reference lesion.

In the benign example, the predicted segmentation achieved a DSC of 0.82 and an IoU of 0.69 relative to the contributor-provided reference mask. In the malignant example, the corresponding values were 0.91 and 0.83, and the predicted subtype was malignant. Figure 6C shows a case with no overlap between the predicted segmentation and reference lesion (DSC=0), while the classification component predicted tumour presence; this case would therefore be discordant under the agreement rule.

#### 4.5.2 Case-level findings in the external series

Tumour detection was recorded for all 11 available cases, and subtype classification was correct in nine: all seven benign cases and two of the four malignant cases (Table 9). These findings are descriptive because the series is small, selected, and entirely tumour-positive.

**Table 9:** Case-level findings for the exploratory external tumour-positive series.

Case	Reference subtype	Tumour detected	Subtype classification
Benign 1	Benign	True	Correct
Benign 2	Benign	True	Correct
Benign 3	Benign	True	Correct
Benign 4	Benign	True	Correct
Benign 5	Benign	True	Correct
Benign 6	Benign	True	Correct
Benign 7	Benign	True	Correct
Malignant 1	Malignant	True	Incorrect
Malignant 2	Malignant	True	Correct
Malignant 3	Malignant	True	Incorrect
Malignant 4	Malignant	True	Correct

## 5 Discussion

### 5.1 Interpretation of key findings

The study evaluated complementary localisation, segmentation, classification, and XAI outputs on the public BTXRD dataset. YOLOv8s-seg achieved an overall box mAP50 of 0.676 and mask mAP50 of 0.668, while ConvNeXt-Tiny produced the strongest binary classification performance under the common-resolution protocol, with an accuracy of 0.879 and ROC-AUC of 0.942. Lesion-size analysis also showed lower tumour-presence sensitivity for small lesions than for large lesions across YOLOv8s-seg and all three CNN classifiers.

The paired metadata analysis indicated that age, dataset-recorded gender, and radiographic projection contributed little additional predictive information beyond the radiograph in the implemented architecture. Small ROC-AUC increases were observed for MobileNetV3-Large and EfficientNet-B3, but test accuracy did not improve. Calibration analysis further showed that the raw fused classifier scores were not well calibrated as probabilities, supporting their use as model scores rather than case-level estimates of diagnostic certainty.

The quantitative XAI analysis showed architecture-dependent spatial correspondence between activation maps and tumour annotations. Mean IoU remained low across all model–method combinations, while the thresholded lesion-overlap measure was higher for MobileNetV3-Large and EfficientNet-B3 than for ConvNeXt-Tiny. Notably, ConvNeXt-Tiny achieved the strongest classification performance but the lowest thresholded lesion-overlap rates, indicating that stronger classification performance did not correspond to closer spatial alignment of the evaluated CAM explanations. These results are descriptive spatial-alignment findings and should not be interpreted as evidence that one model provides more faithful or clinically reliable explanations. Although ConvNeXt-Tiny achieved the strongest classification performance, EfficientNet-B3 retained strong classification performance and showed substantially higher thresholded lesion-overlap rates. EfficientNet-B3 was therefore used in the retrospective agreement analysis.

Classification errors provide additional context for the XAI findings. Smaller lesions were more frequently missed across all three CNN architectures, showing that false-negative predictions were more frequent among smaller lesions. False-positive predictions could not be assessed using tumour-mask overlap because these images contain no reference tumour region. The qualitative error examples further illustrate the distinction between classifier activation and the final classification output. In Supplementary Figure S1A, all three CAM methods show strong activation over the distal foot despite the absence of an annotated tumour, suggesting that normal anatomical or image features may contribute to a tumour-positive response. In Supplementary Figure S1B, the activation maps overlap part of the annotated lesion despite the negative classifier output, showing that spatial activation may still be present when the final score does not produce a tumour-positive classification. These examples support the use of XAI maps as complementary visual information rather than as standalone evidence of classification correctness. Within the dual-component design, this distinction also supports considering the classifier-associated XAI maps alongside the independent YOLOv8s-seg localisation and segmentation outputs. The possible value of combining these outputs during clinical review would require prospective evaluation with clinicians.

The agreement analysis characterised a performance–coverage trade-off rather than an increase in overall test accuracy. EfficientNet-B3 and YOLOv8s-seg agreed on 80.7% of test radiographs, with 93.6% binary accuracy within that agreed subset; 19.3% of cases were discordant and received no consensus output. This retrospective analysis distinguishes concordant from discordant model outputs, but it does not establish the safety or clinical effectiveness of a triage workflow.

The external series offered a limited examination of model behaviour outside the BTXRD test partition, including favourable segmentation examples, subtype errors, and a discordant component output. Its small, selected, all-tumour-positive composition prevents estimation of general external diagnostic performance.

### 5.2 Comparison with prior work

YOLOv8s-seg achieved a three-class diagnostic accuracy of 85.2%, compared with the 71.65% reported by Sen et al. (8); direct comparison is limited by differences in datasets and evaluation tasks. ConvNeXt-Tiny achieved 87.9% binary classification accuracy, while Rajath et al. (9) reported 94% using CT rather than plain radiographs. Recent bone-tumour studies have increasingly incorporated Grad-CAM and related XAI methods (10–13), although quantitative comparison of activation maps with expert tumour annotations remains less common. The present study additionally compares three gradient-based XAI methods against tumour masks and reports an agreement-based analysis using coverage and selective accuracy. Together, these analyses provide a common-dataset comparison of classification, localisation, XAI spatial alignment, and component agreement. Direct performance comparisons with previous studies remain limited by differences in datasets, tasks, and imaging modalities.

These findings should also be considered within the broader methodological literature on medical AI. Reporting guidance such as CLAIM 2024 emphasises transparent reporting of model development, evaluation, uncertainty, and reproducibility (25), while adequate external testing is important for assessing model generalisability beyond the development dataset (26). The calibration findings are consistent with previous evidence that neural-network confidence scores may be poorly calibrated (27). Similarly, the agreement analysis can be considered within the selective-prediction framework, in which predictive performance is evaluated together with coverage (28). Finally, visually plausible saliency maps do not necessarily establish explanation faithfulness (29), and prospective clinical evaluation incorporating human factors remains necessary before conclusions regarding clinical utility can be made (30).

### 5.3 Limitations of the study

The BTXRD dataset is unevenly distributed across anatomical sites and tumour subtypes, which limits the stability of subgroup estimates and may contribute to poorer performance in less represented regions. The dataset was split using unique image identifiers with no image-level overlap between training, validation, and test partitions; however, patient and examination identifiers were unavailable, so patient-level separation could not be verified. The reported estimates should therefore be interpreted as image-level results.

The external analysis was restricted to 11 selected tumour-positive radiographs, comprising seven benign and four malignant cases. The absence of normal radiographs prevented estimation of specificity, false-positive rate, or positive predictive value under representative disease prevalence. Diagnostic labels and masks were used as supplied by the clinical collaborator, without re-annotation or adjudication by the present study team. The series is therefore exploratory rather than an independent external validation cohort.

The agreement analysis represents a single retrospective operating point. Agreement between two models does not establish that a shared prediction is correct, and 19.3% of test radiographs were discordant. Prospective evaluation would be required to determine whether an agreement-based review rule could affect workload or diagnostic outcomes.

Calibration was assessed descriptively on the same held-out test partition used for performance reporting, and no independent recalibration experiment was performed. The observed calibration results therefore should not be assumed to transfer across institutions, patient groups, or acquisition settings.

The XAI analysis was limited to Grad-CAM, Grad-CAM++, and Layer-CAM applied to the image-only tumour logit. Activation-map overlap depends on the selected target layer, normalisation, and thresholding procedure, and spatial overlap does not establish causal faithfulness. Perturbation-based faithfulness tests, stability analyses, and blinded clinician assessment were not performed. The findings are specific to radiographs and may not transfer to other imaging modalities or broader external datasets.

## 6 Conclusions

This study provides a rigorous common-dataset evaluation of complementary localisation, segmentation, classification, and XAI methods for bone tumour assessment on radiographs. Under the controlled common-resolution protocol, ConvNeXt-Tiny achieved the strongest binary classification performance, while YOLOv8s-seg provided complementary lesion localisation, segmentation, and subtype information. The subgroup analyses further showed that smaller lesions were consistently more difficult to detect, while contextual metadata contributed only limited incremental predictive value.

The study also demonstrates the value of evaluating model behaviour beyond classification accuracy alone. Quantitative XAI analysis revealed substantial differences in spatial alignment across architectures, and the EfficientNet-B3–YOLOv8s-seg agreement analysis identified a high-accuracy concordant subset covering 80.7% of the test set, with 93.6% binary accuracy within that subset. Together, these findings show how complementary model outputs can provide a more informative assessment of model behaviour than any single performance measure in isolation, while maintaining a clear distinction between retrospective model evaluation and validated clinical use.

Overall, the results support the value of combining classification, localisation, segmentation, and explainability analyses within a single reproducible evaluation framework for bone tumour radiographs. Larger independent external datasets, patient-level evaluation where identifiers are available, and prospective reader or workflow studies are now needed to determine how these complementary outputs may translate into clinically useful decision support.

## Data Availability

The datasets presented in this study can be found in online repositories. The names of the repository/repositories and accession number(s) can be found below: The BTXRD dataset analysed in this study is publicly available at https://doi.org/10.1038/s41597-024-04311-y. The external clinical case-series dataset comprising 11 anonymised radiographs and corresponding segmentation masks is publicly available at https://github.com/yasirhafeez840/Dr_Shahabuddin_Siddiqui. The source code for the models developed in this study is available at https://github.com/psxdj6/Bone-Tumor-Classification-XAI.
