# Supplementary material for: Comparative evaluation of explainable vision models for bone tumour detection on radiographs
Source: Front Digit Health. 2026 Sep 23;8:1926330. doi: 10.3389/fdgth.2026.1926330 (PMC13642675; doi:10.3389/fdgth.2026.1926330)
Supplement: Supplementary file 1 [file Image1.pdf]

Supplementary Material

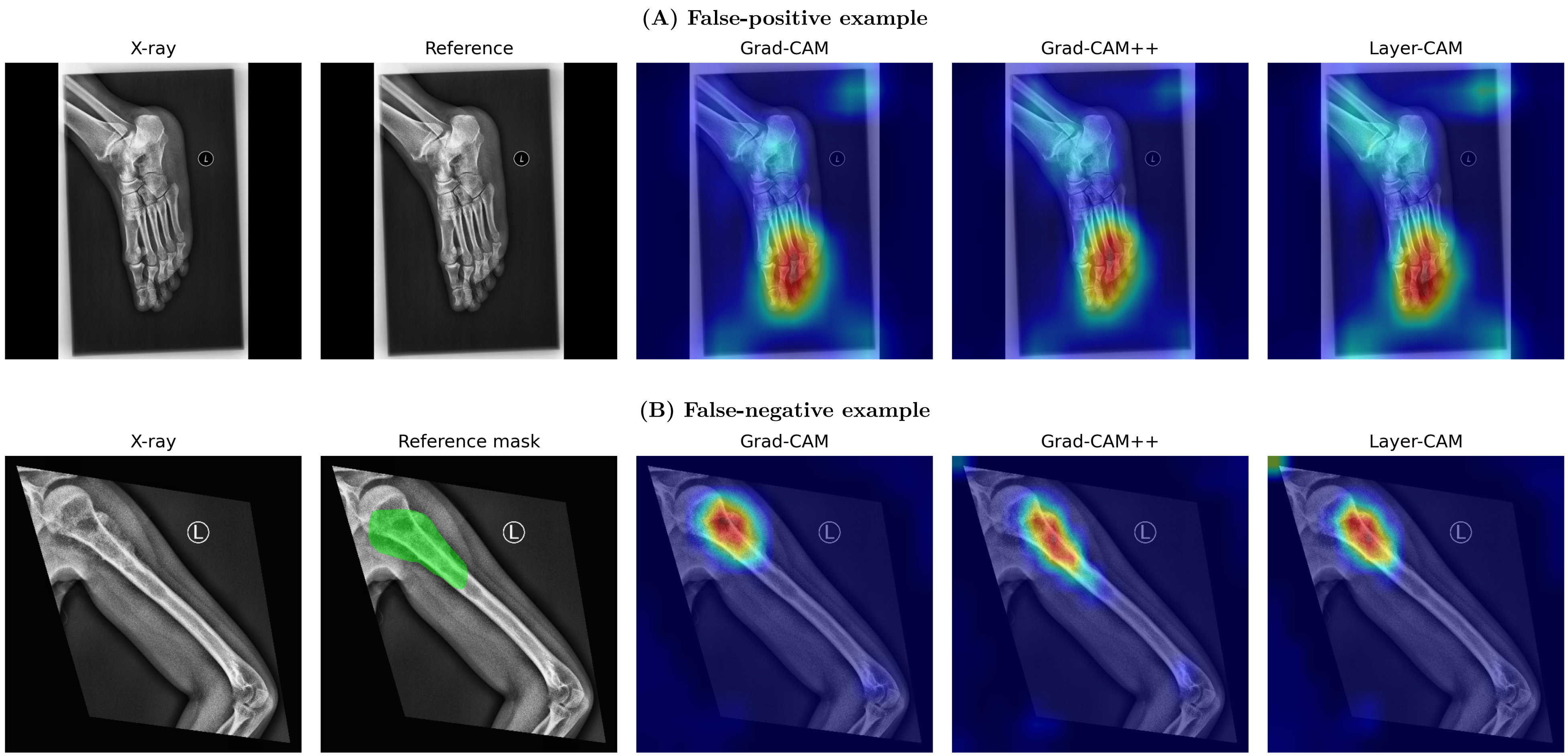

Supplementary Figure S1: Illustrative EfficientNet-B3 classification errors from the held-out test set. (A) False-positive prediction in a no-tumour radiograph. (B) False-negative prediction in a tumour-positive radiograph, with the reference tumour mask shown in green. Grad-CAM, Grad-CAM++, and Layer-CAM activation maps are shown for both cases. The false-positive example was the highest-scoring false positive and the false-negative example was the lowest-scoring false negative according to the image-only tumour score, as described in the Methods.
